# Fungating malignant peripheral nerve sheath tumor arising from a slow-growing mass in the forearm: a case report and review of the literature

**DOI:** 10.1186/s13256-020-02427-4

**Published:** 2020-07-07

**Authors:** Samer Abdel Al, Mohamad K. Abou Chaar, Wafa Asha, Hani Al-Najjar, Maysa Al-Hussaini

**Affiliations:** 1 Department of Orthopedic Oncology, King Hussein Cancer Center, Amman, 11941 Jordan; 2 Department of Surgery, King Hussein Cancer Center, Amman, Jordan; 3 Department of Radiation Oncology, King Hussein Cancer Center, Amman, Jordan; 4 Department of Pathology and Laboratory Medicine, King Hussein Cancer Center, Amman, Jordan

**Keywords:** Malignant peripheral nerve sheath tumor, Sarcoma, Forearm

## Abstract

**Background:**

Malignant peripheral nerve sheath tumor is a rare and aggressive form of sarcoma that arises from a peripheral nerve, mostly in association with neurofibromatosis type 1. Half of the cases were reported in the extremities, with the lungs being the most common site of metastasis. We report a rare case of successful limb salvage surgery performed for a large exophytic malignant peripheral nerve sheath tumor of the right forearm with wide surgical margins followed by split-thickness skin graft and later a flexor carpi radialis tendon transfer to extensor digitorum communis tendon.

**Case presentation:**

A 51-year-old Bedouin Arabic man presented to our institution with an incompletely excised, large, fungating, malignant peripheral nerve sheath tumor occupying most of his right forearm. Staging imaging showed multiple lung nodules. He underwent wide local excision followed by skin graft and tendon transfer as a palliative measure to preserve the function of his dominant limb. The operation was performed without any complications, and the patient had an excellent postoperative result. Afterward, he was started on multiple lines of chemotherapy that failed because of disease progression, and the patient died 7 months after the operation.

**Conclusion:**

Clinicians must consider the possibility of soft tissue sarcoma even in a patient with a small, slow-growing, superficial mass. Furthermore, a wrong open biopsy or nononcological surgical procedure may lead to possible contamination and ultimately a more radical procedure than would have originally been necessary, where this can be prevented by an early referral to a highly specialized sarcoma center.

## Introduction

Malignant peripheral nerve sheath tumor (MPNST) accounts for 5–10% of all soft tissue sarcomas [1]. It is defined as an aggressive and uncommon malignant tumor arising from a peripheral nerve or showing nerve sheath differentiation [2]. Most patients diagnosed with MPNSTs are adults with an age range of 20 to 50 years [3]. Typically, the patient presents with an enlarging mass that might be associated with pain, paresthesia, weakness, or radicular pain [4]. It is well documented that patients with neurofibromatosis type 1 (NF1) have a greater lifetime risk of developing MPNSTs (8–13%) and worse prognosis in comparison with sporadic cases, with 5-year disease-specific survival of 16–38% versus 42–57%, respectively [2, 5–10].

This case report discusses potential approaches to the management of a large fungating MPNST tumor mass in the dominant upper limb of an active middle-aged man with lung metastasis at the time of presentation. Opportunities and challenges in the management of such a case in a specialized cancer center in a low- to middle-income country are shared.

## Case presentation

**Fig. 1:**
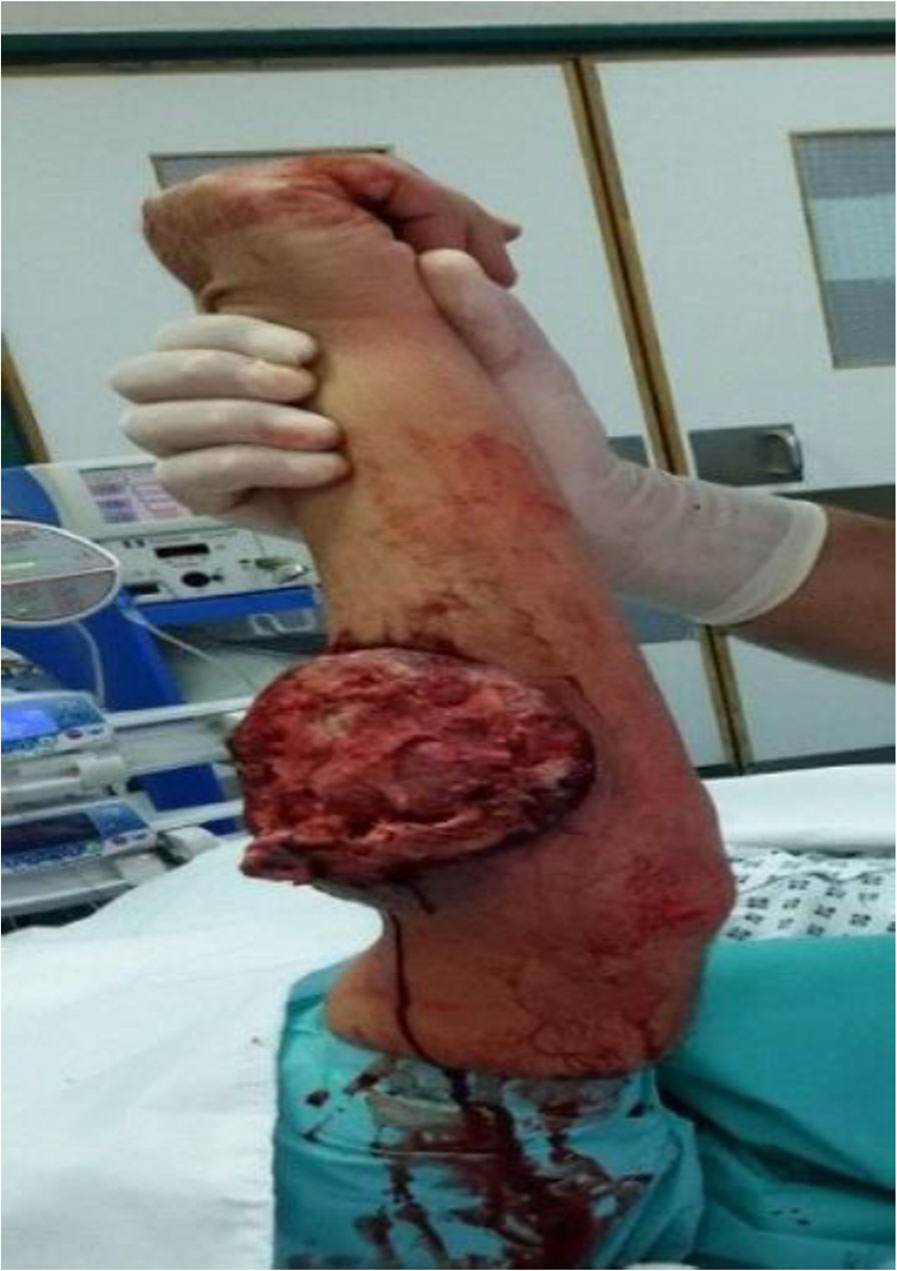
Preoperative photo taken of the fungating tumor after isolating the forearm in preparation for sterile draping. Blood can be seen oozing from the tumor

**Fig. 2:**
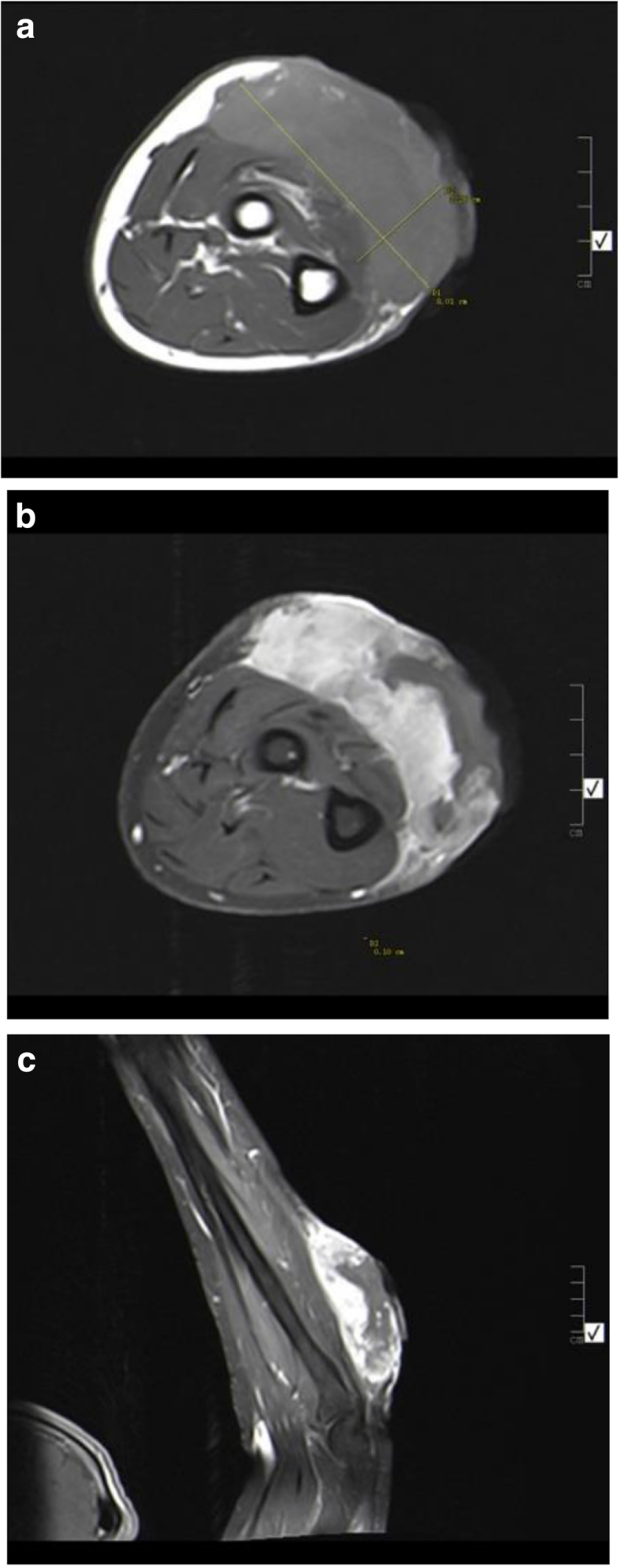
**a** Magnetic resonance imaging (MRI) of the forearm. Axial T1-weighted image shows infiltrative subcutaneous mass involving the proximal ulnar aspect with skin defect and slightly hyperintense signal IIA3. **b** MRI of the forearm. Axial T1-weighted fat-saturated postcontrast image shows significant enhancement with internal necrosis IIA1. **c** MRI of the forearm. Sagittal T1-weighted fat-saturated postcontrast material infusion IIA2

**Fig. 3:**
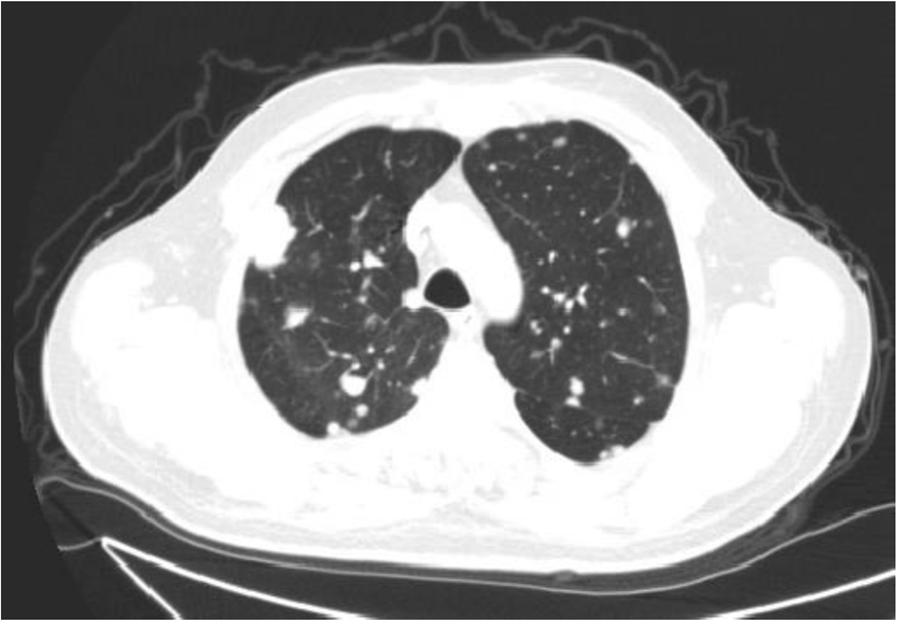
Computed tomographic scan of the chest in transaxial view with lung window showing multiple lung nodules that most likely correlate with metastatic disease

A 51-year-old Bedouin Arabic man with a dominant right hand noticed a small nodule on the proximal dorsal side of his right forearm that had persisted for 18 months. He had not sought any medical advice. He is a known smoker and works as a security guard, has no prior history of alcohol consumption or radiation exposure, was previously diagnosed with depression and receiving oral risperidone 4 mg, and has a positive family history of ocular and breast cancer. When first noticed, the painless nodule was 1.0 × 1.0 cm, but it started to grow slowly over the course of 18 months. It was excised at an outside facility when it reached roughly 4.0 × 4.0 cm because it was thought to be a benign mass. It was removed in pieces using a transverse incision, and pathological examination showed a spindle-cell tumor with features suggestive of MPNST. The patient presented to King Hussein Cancer Center 4 months after the initial excision with an oval-shaped, large, exophytic, fungating, hemorrhagic, firm, and painful mass measuring 10.0 × 15.0 cm at the same site of the previously excised nodule (Fig. 1). At presentation, his vital signs were unremarkable, with a temperature of 36.6 °C, pulse rate of 72 beats/minute, respiratory rate of 18 breaths/minute, blood pressure of 114/76 mmHg, oxygen saturation of 96%, and a pain score of 7/10. His physical checkup revealed power of 3+/5 in his fingers and wrist extension, an intact sensation at the dorsum of the hand with intact median and ulnar nerve function, full range of motion in the elbow and shoulder joints with an intact vascular examination, and no evidence of local lymph node enlargement. Magnetic resonance imaging (MRI) demonstrated a predominantly subcutaneous soft tissue mass at the proximal ulnar aspect of the right forearm with evidence of surrounding muscular fascial involvement and irregularity of the overlying skin, albeit without invasion of the surrounding bony structures (Fig. 2a–c). The mass measured 8.0 × 3.3 cm in the axial dimension and 9.8 cm in the craniocaudal dimension. Whole-body positron emission tomography/computed tomography scan showed multiple, bilateral, variably sized pulmonary nodules consistent with pulmonary metastasis, the largest of which measured 1.6 × 1.4 cm in transaxial dimension adjacent to the right oblique fissure (Fig. 3). The result of a bone scan was negative. A multidisciplinary committee discussed with the patient and his family the treatment plan, stating that his cancer was in stage IV according to the American Joint Committee on Cancer guidelines [11], including starting with chemotherapy followed by surgical excision, but he opted to proceed with the excision first because he could not tolerate the foul smell and severe pain and was concerned that he might develop an infection if he was left untreated. Also, it was discussed that pulmonary metastasectomy was impossible to achieve while maintaining functional lung capacity. On admission, the patient had a hemoglobin level of 13.7 g/dl, white blood cell count of 10.2 × 10^3^/μl, platelet count of 234 × 10^3^/μl, albumin of 4 g/dl, creatinine of 0.7 mg/dl, and normal values for the rest of the liver and kidney function tests.

**Fig. 4:**
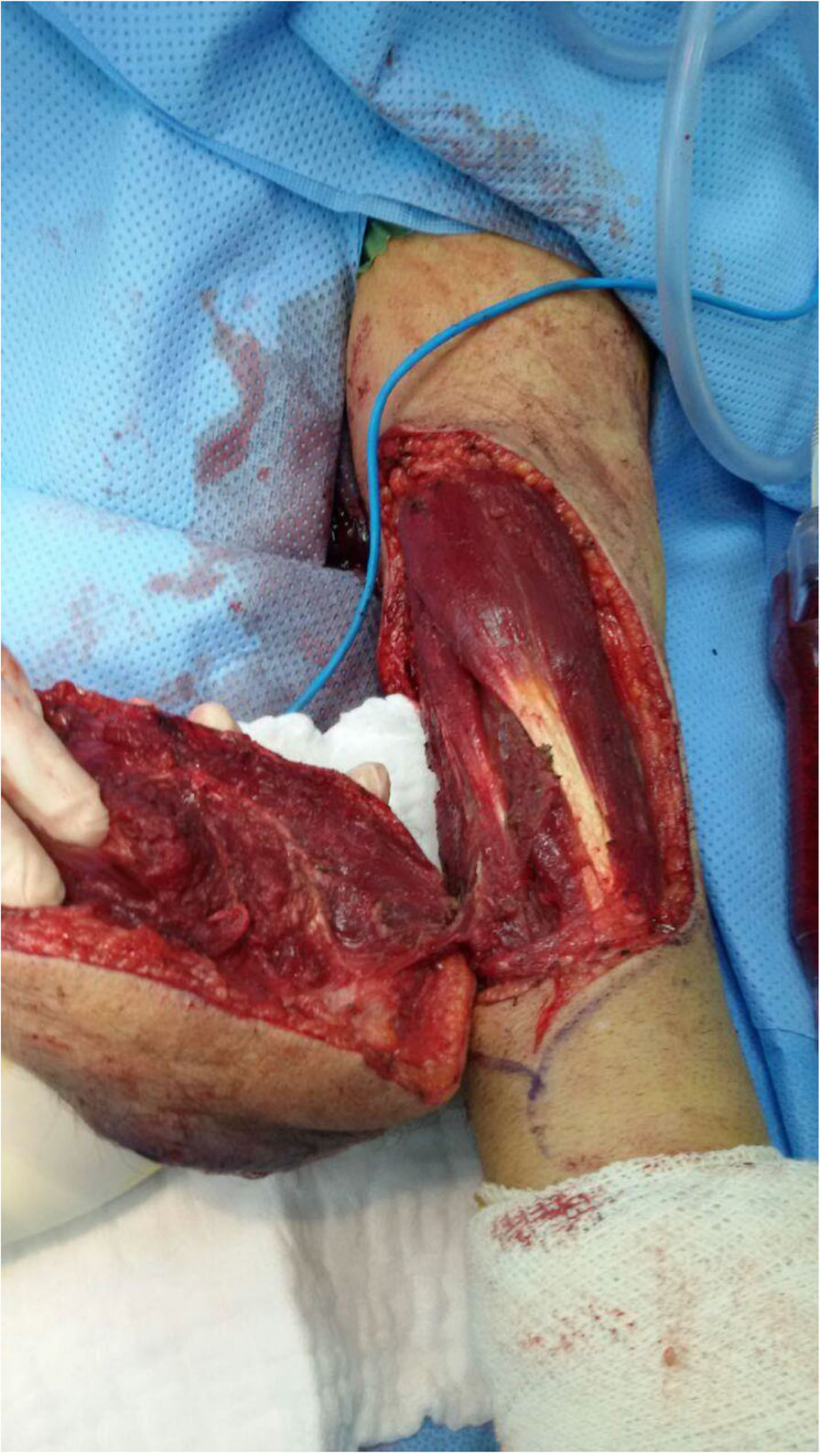
Intraoperative photo showing complete *en bloc* resection of the mass with a safety margin from the extensor digitorum communis muscle

**Fig. 5:**
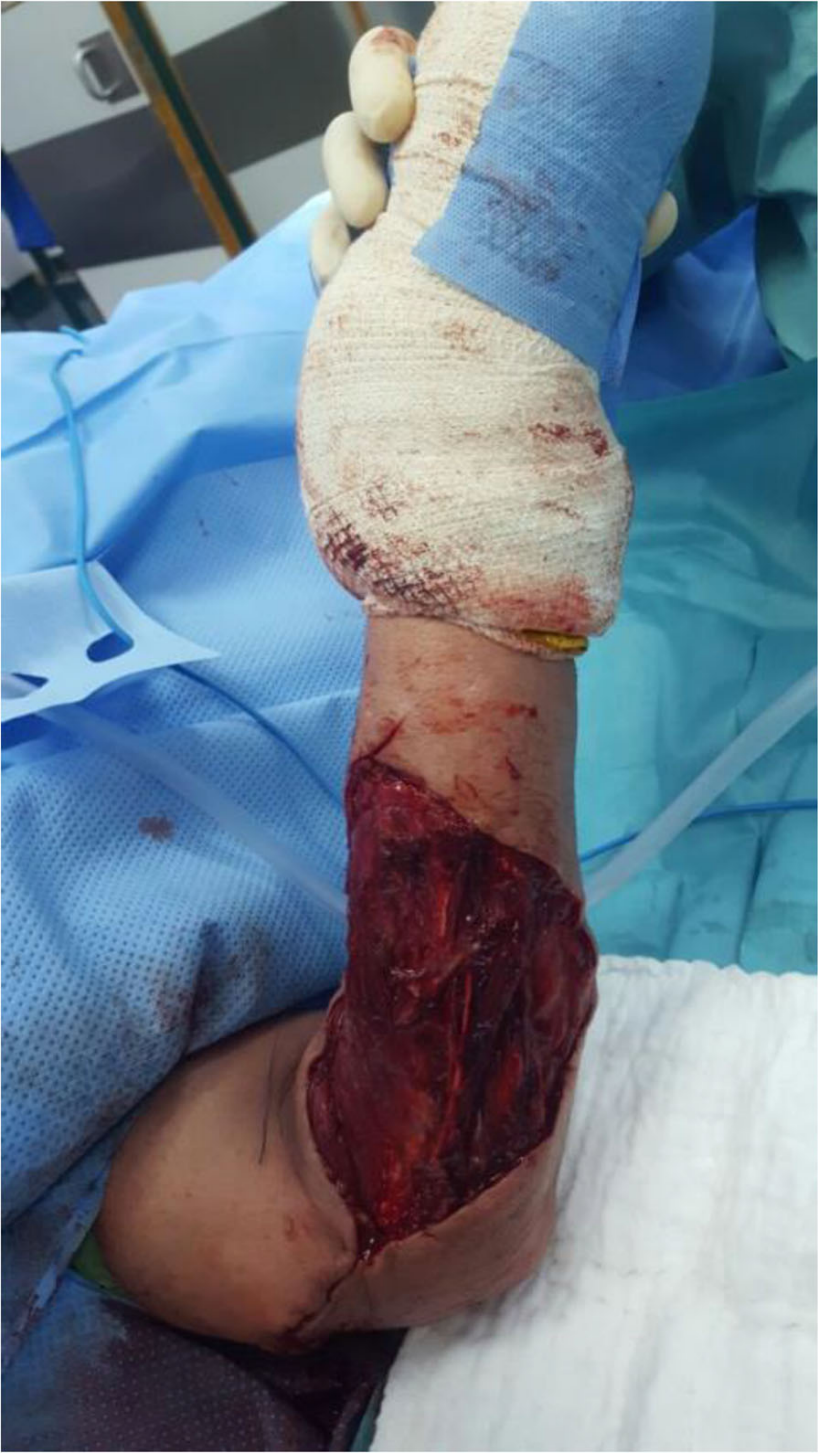
Intraoperative photo showing muscle approximation that was done to cover the exposed tendon, as well as an approximation of the skin using the purse-string technique

**Fig. 6:**
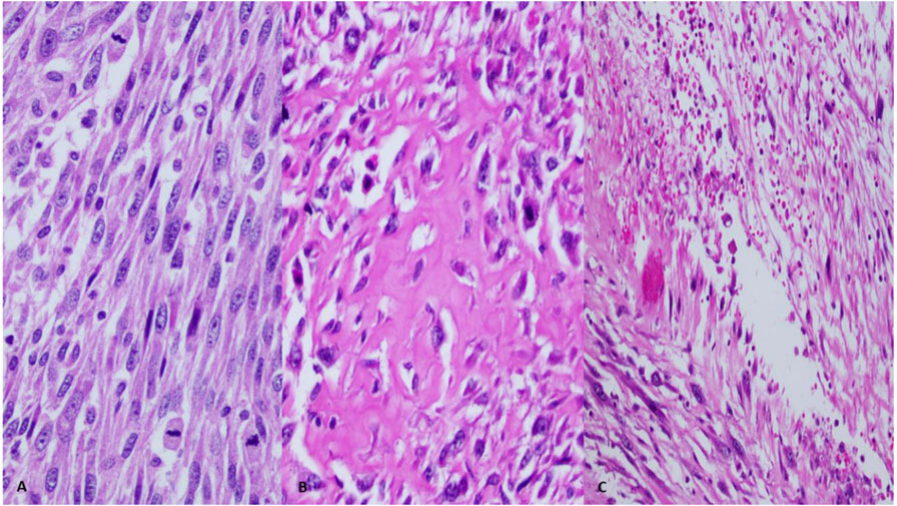
Microscopic examination of the tumor. **a** There is a proliferation of spindle cells with abundant mitotic figures. **b** Heterologous elements in the form of osteoid are seen, laid directly by the tumor cells. **c** Palisading necrosis is identified. All images are stained with hematoxylin and eosin. Original magnification × 40

**Fig. 7:**
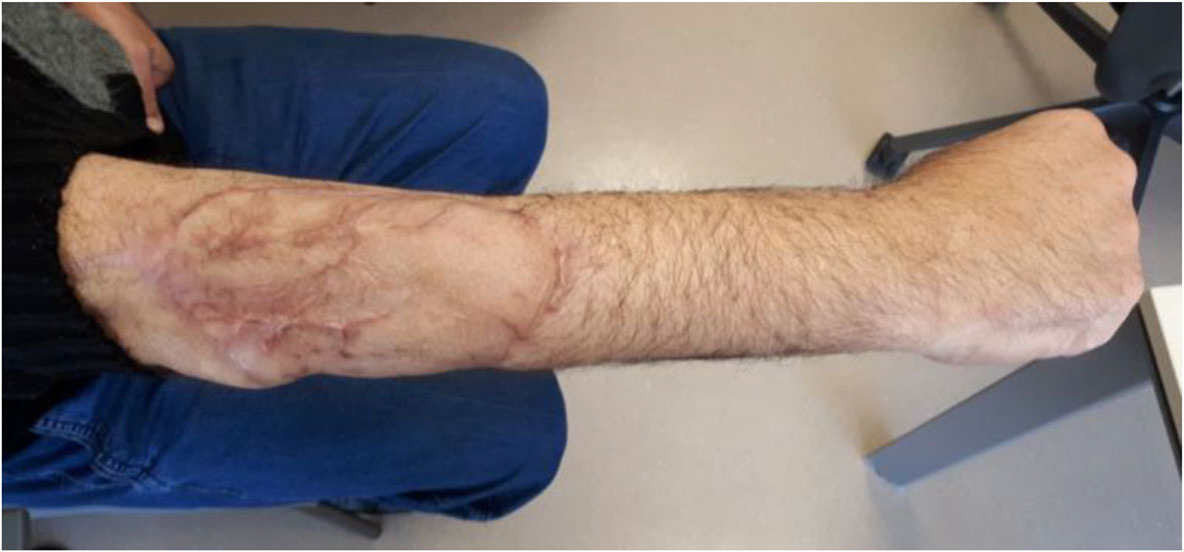
Right forearm photo taken in the clinic 1 month after the operation, showing completely healed and well-taken graft with the elbow in full extension

With the aid of a bloodless field using a tourniquet without exsanguination, we outlined a wide local excision about 3 cm away from the tumor. An elliptical incision was made while carefully inspecting the soft tissue mass to avoid any iatrogenic dissection through the tumor. Multiple frozen sections were sent during the operation from the deep proximal and deep distal ends, and the result was negative for malignancy. The mass was taken *en bloc* with a safety margin from the extensor digitorum communis muscle (EDCM) (Fig. 4). An approximation of the muscles was done to cover the exposed tendon, as well as undermining and approximation of skin (Fig. 5). This was followed by vacuum pressure dressing and physiotherapy. After 10 days, the pathology report was issued with the final diagnosis of grade 3 MPNST with heterologous bony elements and negative resection margins (Fig. 6). The patient was then staged for another procedure for both flexor carpi radialis tendon transfer to extensor digitorum communis tendon and soft tissue defect coverage with a split-thickness skin graft harvested from the ipsilateral thigh. The patient was splinted for 1 month, and he was seen every 2 weeks to check for the skin graft until it was completely healed (Fig. 7). Two months after the procedure, and on a biweekly basis, physiotherapy and occupational therapy were resumed after the removal of the splint. The patient regained his full range of elbow motion as well as most of his finger extension.

The patient was then started on palliative intravenous chemotherapy (two cycles of 3600 mg ifosfamide and 200 mg etoposide daily for 5 consecutive days in each cycle) and radiotherapy to his lung metastasis (50 Gy/eight fractions), which all failed because of disease progression. The patient was planned for single-agent doxorubicin, but he developed respiratory failure type 2 and elected a “do not resuscitate” status, so he was referred for palliative care. The patient died at home 7 months after the operation, and an autopsy was not done, because it is only indicated in cases of homicide or upon family request in Jordan.

## Discussion

We present a rare case of a solitary, slow-growing mass that persisted for 18 months, which is a nontypical feature of sarcomas, which have very rapid and aggressive behavior. MPNST (previously known as neurogenic sarcoma, neurofibrosarcoma, or malignant schwannoma) is a relatively rare malignant tumor, accounting for 5–10% of all soft tissue sarcomas with an incidence rate of 0.001% in the general population and 4.6% in patients with NF1 [7]. Although NF1 gene inactivation and loss of neurofibromin expression characterize the majority of MPNST cases [12], biallelic NF1 loss is insufficient for malignant transformation, and mutations in TP53, CDKN2A, EGFR, and SUZ12 have all been reported as secondary cooperating mutations facilitating malignant progression [13–17].

The age range at presentation is 20 to 50 years. MPNST is most commonly located in the extremities (45%), followed by the trunk (34%) and the head and neck areas (19%), as reported by Stucky *et al.* [3]. However, Kim *et al*., who analyzed a large cohort of patients over 30 years, concluded that peripheral nerve tumors have a higher prevalence in the upper limb than in the lower limb [18]. Gosk *et al.* reviewed 94 cases of peripheral nerve tumors, only 1 of which was diagnosed as MPNST, and the patient underwent arm amputation [19].

Due to the rapid pace of tumor progression in MPNST cases, most patients seek medical advice when the tumor increases in size or when it starts to cause neuropathic symptoms ranging from paresthesia to radicular pain [4]. Stucky *et al.* reported metastasis in 67 of 175 patients, either at the time of presentation or in follow-up, with the lung being the most common site (55%), followed by intra-abdominal visceral and osseous metastases [3]. Similarly, Sordillo *et al*. reported the lung as the most common site of distant metastasis, followed by the liver, the peritoneum, and subcutaneous tissue [2].

MPNSTs can resemble benign tumors, both histologically and radiologically. Demir *et al*. suggested that the diagnosis is easily made in patients without NF1 who present with a palpable mass and pain; conversely, the diagnosis is frequently delayed in patients with NF1 due to potential misdiagnosis of these lesions as classical neurofibroma and/or plexiform neurofibroma [20]. To ensure early diagnosis of MPNSTs, MRI evaluation and biopsy should be performed immediately when malignancy is suspected [21, 22]. However, plexiform neurofibromas and MPNSTs have similar MRI findings, including areas of low to intermediate signal intensity on T1-weighted sequences and high signal intensity on T2-weighted sequences with heterogeneous enhancement. Wasa *et al.* reported that patients with MPNSTs generally have giant masses, peripheral enhancement patterns, perilesional edema-like zones, and intratumoral cystic lesions [23]. Histologically, MPNSTs show a fasciculated growth pattern with hyperchromatic spindle cells. Nuclei are elongated and wavy, and perivascular hypercellularity is often noted. Marked mitotic activity and geographic tumor necrosis are also commonly present [3].

The prognosis of MPNSTs is poor, especially for tumors that cannot be resected. Compared with other soft tissue sarcomas, MPNST has the highest risk of sarcoma-specific death [18, 19]. Anghileri *et al.* reported that large tumor size, truncal location, and positive surgical margins were significant factors predicting local recurrence, whereas recurrence at presentation, tumor size, and tumor grade are significant factors for distant metastases [24]. This is similar to findings reported by Stucky *et al.* [3]. It should be noted that both studies identified local recurrence, size > 5.0 cm, and truncal location of the primary tumor as being associated with adverse survival. Additionally, Stucky *et al*. commented that high tumor grade also affects survival, keeping in mind that patients with NF1-associated MPNST have a significantly worse disease-free survival than sporadic cases [2, 3, 5–10].

The mainstay of treatment is an aggressive surgical approach with negative margins (R0 resection) followed by adjuvant radiotherapy for tumors with high grade, large size, and positive margins and the addition of chemotherapy for those who can tolerate it [3]. Chase indicated that limb salvage surgery can be attempted if blood supply is adequate for vascular anastomoses or grafts. The residual elements of the extremity can be salvaged to serve their original intended purpose and keeping in mind assessing the importance and likelihood of success of limb salvage in relationship to the patient’s well-being and other possible disabilities (age, the dominance of hand, loss of a contralateral extremity, blindness, intended future occupation, and patient’s personal needs) [25]. This was one of the major reasons why we opted to perform limb salvage surgery for a foul-smelling and fungating MPNST in a metastatic patient. If neoadjuvant treatment is approached, National Comprehensive Cancer Network guidelines state that a combination of etoposide/ifosfamide should be given for stages II and III [26]. It should be noted that results of an ongoing multicenter phase II trial (NCT00304083) that studies combination chemotherapy with doxorubicin, etoposide, and ifosfamide in unresectable (stages III–IV) adult MPNST are awaited. The use of adjuvant doxorubicin-based cytotoxic chemotherapy for MPNST has also been debated, with several studies failing to show a survival benefit of chemotherapy in the treatment of MPNST [9, 27, 28]. Further understanding of the genomics, epigenetics, signaling pathways, and metabolic alterations in MPNST, coupled with a better comprehension of the cross-talk between tumor and surrounding microenvironment, will lead to more novel combinations of targeted therapies that have increased efficacy and specificity in these tumors.

The difficulties faced in treating these unusual cases in the setting of metastatic disease are divided between focusing on systemic versus local surgical treatment. If a local surgical excision is pursued, which might interrupt the systemic treatment in some cases, what will be the better form of local surgical intervention: limb salvage or palliative amputation? Such issues remain debatable, without a strong evidence supporting any method. We elected to proceed with limb salvage surgery to achieve a functional rather than survival outcome, which seems more important in the setting of widespread metastasis and poor prognosis.

## Conclusion

This case report summarizes the surgical forearm preservation of a rare presentation of MPNST where a solitary, nonspecific, small, superficial, slow-growing mass encountered in the limbs should alert physicians to consider the possibility of sarcoma and use a careful approach. Unfortunately, in the setting of low- to intermediate-income countries with limited resources, patients are frequently referred to tertiary cancer centers when the disease has already rapidly progressed locally, most likely due to possible contamination by a wrong open biopsy or nononcological surgical procedure. We believe that, sharing and publishing our experience in managing challenging sarcoma cases will better enhance the understanding of how to approach and treat similar cases.

## Data Availability

Not applicable.

## Abbreviations

EDCM: Extensor digitorum communis muscle
MPNST: Malignant peripheral nerve sheath tumor
MRI: Magnetic resonance imaging
NF1: Neurofibromatosis type 1
